# Amount, not strength of recollection, drives hippocampal activity: A problem for apparent word familiarity‐related hippocampal activation

**DOI:** 10.1002/hipo.23031

**Published:** 2018-11-08

**Authors:** Andrew R. Mayes, Daniela Montaldi, Adrian Roper, Ellen M. Migo, Taha Gholipour, Alex Kafkas

**Affiliations:** ^1^ Memory Research Unit, School of Biological Sciences, Division of Neuroscience & Experimental Psychology University of Manchester Manchester United Kingdom; ^2^ Epilepsy Center The George Washington University Washington DC

**Keywords:** familiarity, hippocampus, recall, word stimuli

## Abstract

The role of the hippocampus in recollection and familiarity remains debated. Using functional magnetic resonance imaging (fMRI), we explored whether hippocampal activity is modulated by increasing recollection confidence, increasing amount of recalled information, or both. We also investigated whether any hippocampal differences between recollection and familiarity relate to processing differences or amount of information in memory. Across two fMRI tasks, we separately compared brain responses to levels of confidence for cued word recall and word familiarity, respectively. Contrary to previous beliefs, increasing confidence/accuracy of cued recall of studied words did not increase hippocampal activity, when unconfounded by amount recollected. In contrast, additional recollection (i.e., recollecting more information than the word alone) increased hippocampal activity, although its accuracy matched that of word recall alone. Unlike cued word recall, increasing word familiarity accuracy did increase hippocampal activity linearly, although at an uncorrected level. This finding occurred although cued word recall and familiarity memory seemed matched with respect to information in memory. The detailed characteristics of these effects do not prove that word familiarity is exceptional in having hippocampal neural correlates. They suggest instead that participants fail to identify some aspects of recollection, misreporting it as familiarity, a problem with word‐like items that have strong and recallable semantic associates.

## 1 INTRODUCTION

Recognition of previously encountered stimuli depends on two kinds of memory: recollection and familiarity. Recollection memory leads to stimulus cued recall of details associated with the stimulus from a previous encounter. Recall of these details usually confirms that the stimulus has been encountered before. Recollection is supported by storing stimulus‐study‐context detail associations in a pattern separated way so that similar associations are represented in a more neurally distinct way (Norman & O'Reilly, 2003). Some dual process views of recognition propose that such pattern separation is optimally performed by the hippocampus but not by other medial temporal lobe (MTL) regions (for a discussion, see Montaldi & Mayes, 2010). In contrast, familiarity memory involves feeling that one has previously encountered a stimulus without recollecting any previously encountered details associated with it. Dual process views posit that familiarity is supported by storing stimulus representations so as to amplify their similarities to other stimulus representations, which enables a global matching process to mediate familiarity when the stimulus is later encountered again (Norman & O'Reilly, 2003).

Many have proposed that familiarity depends on the perirhinal cortex (PRC), but not on the hippocampus (for a contrary view, see Wixted & Squire, 2010). Montaldi and Mayes (2010) argued for a variant of the dual processing view according to which, within the MTL, object‐related familiarity depends on the PRC, whereas context‐related familiarity depends on the parahippocampal cortex (PHC). This view implies that each MTL structure's function depends not only on its inputs, which determine *what* it processes, but also on its cytoarchitectonics, which determines *how* it processes its inputs. In turn, the structure's outputs may determine how its processed products are further processed by other brain structures. Thus, the neocortical PRC and PHC support familiarity processing, whereas the archicortical hippocampus supports recall/recollection processing. These two kinds of processing are performed on distinct inputs and the processed products are sent to partially distinct extra‐MTL structures (for empirical evidence, see Kafkas et al., 2017).

With functional magnetic resonance imaging (fMRI) studies, this view predicts that recollection should modulate hippocampal activity, but stimulus familiarity memory should not. In contrast, familiarity should influence PRC or PHC activity depending on the kind of stimulus involved (Kafkas et al., 2017). Provided other routes are available for transmitting object‐related and context‐related information to and from the hippocampus, this view also predicts that recollection of object‐context associations should not modulate activity in the PRC and PHC. Equivalently, selective recollection and familiarity deficits should occur following hippocampal and PRC/PHC lesions, respectively.

However, it has been suggested that findings, whether about fMRI or lesion effects, are untrustworthy because recollection and familiarity are confounded with recognition memory strength. This is because recollection is typically related to strong recognition, whereas familiarity is often related to weaker recognition (see, e.g., Squire, Wixted, & Clark, 2007; Wixted & Squire, 2010). Recognition strength is defined operationally as the accuracy of recognition (hit rate as a proportion of hit rate plus false alarm rate), which is reasonably well predicted by participants' confidence that their recognition is accurate.

There is considerable fMRI evidence examining whether encoding leading to subsequent familiarity and familiarity itself at test modulate activity in the hippocampus. The great majority of this evidence indicates that familiarity has no effect whereas recollection does (e.g., Montaldi, Spencer, Roberts, & Mayes, 2006; Ranganath et al., 2004; Yonelinas, Otten, Shaw, & Rugg, 2005; for a review, see Montaldi & Mayes, 2010). However, due partially to the practical difficulty of doing so, hardly any of these studies managed to control for recognition strength (Montaldi et al. [2006] was for a while the only exception). This has led some researchers to dispute that the studies actually do indicate that the hippocampus does not help mediate item familiarity (e.g., Smith, Wixted, & Squire, 2011). Montaldi et al. (2006) used a familiarity‐only variant of the remember/know procedure (see also Mayes, Montaldi, & Migo, 2007; Migo, Mayes, & Montaldi, 2012), in which participants indicated confidence in familiarity on a scale from 0 to 3 but did not attempt to recollect effortfully, although reporting it when it spontaneously occurred. As indicated above, this enabled them to match strong familiarity (level 3) and recollection with respect to recognition accuracy as well as to determine the effects of increasing familiarity strength (accuracy). With this procedure, it has been shown that increasing strength of familiarity does not change hippocampal activity, and recollection activated the hippocampus more than even equally accurate familiarity for scenes (Montaldi et al., 2006) and objects (Kafkas & Montaldi, 2012). More recently, Kafkas et al. (2017) have replicated these effects with scenes and objects and shown that the effects extend to nonfamous faces.

Even if the MTL structures play different roles in familiarity and recollection, it still remains unresolved whether these neural differences relate to the different recall versus nonrecall processes underlying these two kinds of memory, the additional memory information involved in recollection (stimulus plus associated study context details) versus familiarity (just stimulus), or both of these. This important question has not been previously addressed and it was the focus of the present fMRI study. To achieve our aim, in two separate tasks, we compared a form of cued recall to familiarity. In the cued recall task, participants were cued with part of each studied stimulus to recall only that stimulus, whereas in the other task, familiarity was assessed for another set of word stimuli. This comparison aimed to match the amount and kind of information in recollection and familiarity memory so that only the recall/nonrecall processes differed. We also compared confidence in memory ratings for cued recall of words alone and word familiarity memory. We wished to determine whether increasing recognition accuracy of word recall and familiarity modulated activity in MTL structures, particularly the hippocampus, in the same or different ways. Further, we hoped to match recognition accuracy for cued recall and familiarity at a high level so as to address the recognition strength criticism (Squire et al., 2007). By matching accuracy and amount of information in memory, our aim was to determine whether the cued recall versus no‐cued‐recall difference between familiarity and recollection alone is sufficient to produce processing differences in structures, such as the hippocampus.

Another important aspect of our cued recall task was that we asked participants to indicate when, following cueing, they spontaneously recollected more than the stimulus alone. The aim was to determine to what extent the hippocampus is sensitive to increases in confidence/accuracy or to increases in amount recalled when accuracy remained the same. Specifically, we explored (a) whether increasingly accurate cued recall of only the stimulus activated the hippocampus progressively, (b) whether this only happened when more information was recollected even when this did not increase recognition accuracy, and (c) whether more accurate familiarity did not activate the hippocampus more.

Although there is good evidence that hippocampal activity increases as the amount of information recollected increases (e.g., Rugg et al., 2012), these changes may well be accompanied by similar ones in recognition accuracy. The reported change in hippocampal activity may, therefore, reflect increasing recognition accuracy alone rather than an increase in the amount recollected alone. However, there is preliminary evidence against this interpretation. Qin et al. (2011) found that encoding activity in extra‐MTL but not the MTL structures, including the hippocampus, correlated with subsequent recollection confidence (which correlated with accuracy) for just verbal scene gist, whereas recollecting more information from the scenes correlated with hippocampal and PHC activity. These effects seemed to be statistically independent of each other. However, some caution is warranted because, apart from the need for replication, the generalizability of these findings to retrieval itself and other kinds of stimuli remains uncertain. Also, focusing on the neural independence of a subjective memory measure, gist confidence, an objective memory measure, and the amount of scene details remembered, Qin et al. (2011) failed to measure the correlation between two key objective measures: gist memory accuracy and detail memory amount. Most important, in this prior study, the measure of gist recollection could well have been contaminated with increasing amounts of information recollection as confidence increased. The design of the present study allowed us to address this important issue in a properly controlled way for the first time.

Our final aim was to determine whether familiarity‐related effects with words were similar to those we have previously found for pictorial stimuli in showing no hippocampal involvement (see, e.g., Kafkas et al., 2017). The comparison was potentially strong because in both cases a modified form of the remember/know procedure that matched familiarity and recollection recognition accuracy was used (e.g., Kafkas et al., 2017; Kafkas & Montaldi, 2012). The issue is of particular interest because Smith et al. (2011) found a different pattern of results when they examined familiarity and recollection supporting word recognition. They used a somewhat different procedure in which participants made recognition confidence judgments, followed by recollection or familiarity judgments when words were recognized. This indicated that when familiarity and recollection recognition confidence and accuracy were matched, there was an equal level of hippocampal activation, which was greater than that found with less confident and accurate familiarity. Although other studies have not found that familiarity activated the hippocampus increasingly as a function of word recognition confidence, they did not succeed in matching familiarity and recollection recognition accuracy/strength (e.g., Yonelinas et al., 2005; for a discussion, see Kafkas & Montaldi, 2012; Montaldi & Mayes, 2010).

In outline, the first aim of the present study was to examine whether hippocampal activity is modulated by increasing recollection accuracy alone, increasing amount of information recollected alone, or both. This understanding will allow us to consider the role of the hippocampus in recollection/cued recall and/or familiarity when information in memory as well as recognition accuracy is matched. It will also help us to consider whether any hippocampal differences between recollection and familiarity relate to processing, amount of information in memory, both, or neither. Finally, we will also examine the amount of hippocampal activity generated when recognition accuracy of cued recall, additional recollection, and familiarity are high and similar.

## 2 METHODS

### 2.1 Participants

Informed consent was obtained from 38 right‐handed healthy volunteers who attended a short preselection 10‐min session completed in a mock scanner for which they were paid a pro rata amount. This short session was used as a training session for the main fMRI study (see section 2.3) and in order to familiarize the participants with the scanner environment. From this sample, 21 participants were admitted to the main fMRI experiment, based on their ability to understand the different parts of the study, their willingness to take part, and their likely ability to successfully undergo an fMRI session. Furthermore, in this training session, the selected participants were able to spread their responses across the entire rating scale in the two memory tasks and their memory performance was above chance levels. All participants were native English speakers, reported no neurological or psychiatric disorders, and had normal or corrected‐to‐normal vision (with contact lenses). Data from three participants were excluded from the main fMRI analysis due to a technical problem affecting fMRI acquisition, while one more participant was excluded due to exclusive use of R3 or F3 responses (i.e., strong recollection and strong familiarity responses) in the memory tasks. The remaining 17 participants (10 males) had a mean age of 24.7 (*SD* = 3.6). All study procedures followed in this study were approved by the National Research Ethics Service and participants were paid £20 per session after completing the fMRI experiment.

### 2.2 Stimulus materials

A total of 300 word stimuli (nouns and adjectives) were used across all parts of the experiment, 120 of which were used in the familiarity task, and another 180 in the word stem cued recall task (see section 2.3). The words were selected from the Spontaneous Completion of Three‐Letter Word Stems Database (Migo, Roper, Montaldi, & Mayes, 2010) taking into account the spontaneous word stem completion frequency for each word. Specifically, the 120 words in the familiarity task were between 4 and 11 letters long, had a mean concreteness rate of 538.76 (*SD* = 63.84), a mean imageability rate of 529 (*SD* = 58), and a mean printed familiarity rate of 458.53 (*SD* = 76.33) on the Medical Research Council Psycholinguistic database (Wilson, 1988). Similarly, in the cued recall task, 90 words, between 4 and 9 letters long, were used as target words (studied at encoding). Importantly, spontaneous generation of each word from its corresponding stem was low with a mean frequency of 0.63% (*SD* = 0.85) as determined by previous pilot work with a different sample of 80 participants (Migo et al., 2010). Finally, an additional sample of 90 words of the same three‐word stems with the target words was used as foils in the forced‐choice recognition task.

### 2.3 Procedure

#### 2.3.1 Preselection and training

**Figure 1:**
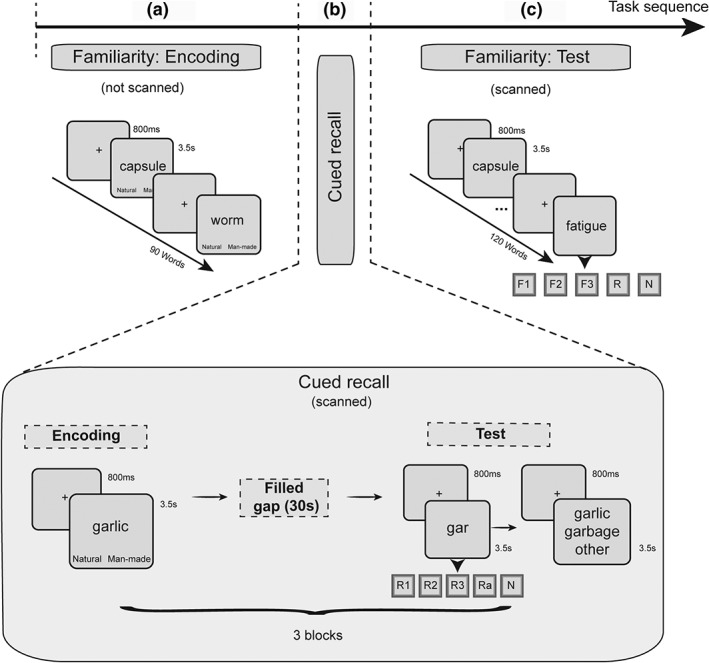
Design of the experiment and sequence of the tasks. Each session began with the familiarity encoding task (a), followed by the cued recall task (b), and finished with the familiarity test task (c). Only (b) and (c) periods were scanned and fMRI data were only analyzed for the test periods in the familiarity and the cued recall tasks

Every participant underwent a short training session within a mock (T0) MR scanner within the University of Manchester campus a few days (1–7 days) prior to participating in the main fMRI experiment. The training session resembled all the different parts of the main study (using different stimuli). Before participating in the study, each participant was individually trained on the procedures followed throughout the experiment, and an outline of the sequence of the different tasks in the present study was presented. This outline is illustrated in Figure 1 (see also Supporting Information). An important aspect of the pre‐experimental training phase was for the participants to understand the difference between familiarity (F) and recollection (R) responses at test (see Supporting Information for instructions), as well as the rating scales used for F and R (see 2.3.2, 2.3.3 and Figure 1). At the end of the training phase, participants were asked to explain the sequence of the tasks and describe what they would be asked to do in each part of the experiment inside and outside the scanner. They also had the opportunity to ask questions and provide examples of experiencing familiarity and recollection in their lives. A reminder of the sequence of the main experiment was also shown to each participant on the day of the fMRI session before starting the experiment.

#### 2.3.2 Word familiarity task

Extensive pilot work had established that to obtain good levels of cued recall and a range of confidence judgments, it was necessary to have a longer study‐test delay for familiarity judgments than cued recall/recollection judgments. Also, although participants were carefully and extensively trained, as both the cued recall/recollection and the familiarity instructions were complex, we considered it appropriate not to intercalate the two tasks (i.e., cued recall and familiarity). This was to avoid any confusion to the participants from having to perform the two tasks in alternating blocks. For these two reasons, the cued recall/recollection task was performed earlier than the familiarity test task in all sessions without counterbalancing across participants.

The sequence of the experimental tasks during the testing session is presented in Figure 1. On the day of the fMRI session, before entering the scanner, participants encoded a series of 90 word stimuli about each of which they were asked to make man‐made versus natural decisions (Figure 1a). Each stimulus was presented for 3.5 s and was preceded by a central fixation point lasting for 800 ms (total block duration: 387 s). Participants were instructed to provide their decision within the word presentation time window. The memory test part of this task (Figure 1c) was completed in the scanner, always after the cued recall task (three encoding and test blocks; see Figure 1b and 2.3.3), and was separated into two blocks of stimuli. During the memory test task, a total of 120 words (90 from the encoding phase; total block duration: 608.5 s) were presented and, using the familiarity‐only procedure (Mayes et al., 2007; Montaldi et al., 2006), participants were asked to focus on making familiarity decisions using three levels of increasing familiarity (F1 = weak, F2 = moderate, and F3 = strong familiarity) or, although asked not to try to recollect, to report any spontaneous recollections (R) that occurred anyway, or to report any new (N) words. In the case of R responses to words, participants were instructed to report a recollection if a stimulus spontaneously brought to mind additional information associated with it from the time of encoding. The content of the recollection may have been a thought while studying the word at encoding, the position of the word in the study list (e.g., the very first/last word), or any other information additional to the presented word associated with the study episode. The allocation of the stimuli as old or new was freshly randomized for each participant. Null events (central fixation) were intermixed with the stimulus trials across the memory test period and were used as the implicit baseline. Responses were collected using a special MR‐compatible response box and participants used both hands to choose one of the five response choices (F1, F2, F3, R, and N). Three fingers from one hand were used for the familiarity rating while two fingers of the other hand were used for the extra two responses. The allocation of the familiarity responses to the left or the right hand was counterbalanced across participants.

#### 2.3.3 Cued recall task

Both encoding and test phases of the cued recall task were completed entirely inside the MRI scanner (Figure 1b), but only retrieval data were analyzed and are reported in the present article. The cued recall task, therefore, was started after placing each participant in the MRI scanner and after the T1 image acquisition from each participant (i.e., ~15 min after the familiarity encoding block). Similar to the familiarity encoding procedure presented earlier, but inside the scanner, participants encoded series of words, providing a man‐made versus natural decision about each word within a presentation period of 3.5 s per word preceded by a central fixation point lasting for 800 ms. Three blocks of 30 words each (i.e., a total of 90 words) were presented in this way, with each block followed by a memory test (Figure 1b). There was then a filled interval of 30 s between encoding and test in each block during which participants were asked to count backward from a given number in steps of 6, 7, 8, or 9 numbers. The use of three study‐test cycles in this task (as opposed to one study and test block) was dictated by pilot work showing that this procedure was needed to provide adequate cued recall performance. Using a novel cued recall‐only procedure, during each of the three memory test sessions, participants were presented with three‐letter word opening stems, matching those from the preceding encoding block, and were asked to recall (recollect) the studied word without trying to recollect anything associated with the word at encoding unless spontaneously. Participants were also asked to refrain from guessing or generating random words for each stem.

Critically, the participants were asked to indicate at the same time how confident they were that the recollected word came from the study episode using a 3‐point scale choosing between weak recollection (R1), moderate recollection (R2), and strong recollection (R3). Two additional responses were provided, one for reporting failure to recall the word at all (N) and another one for reporting additional (noncriterial) recollections (Ra). An Ra response indicated that not only was a specific word recalled from the stem, but also that other additional details that were associated with it during the study episode were also recollected. Each stem was presented for 3.5 s, preceded by a central fixation point of 800 ms, and participants were asked to provide an answer within the stem presentation period, while the response options were visible at the bottom of the screen. As with the familiarity task, null events (central fixation) distributed across the memory test period were used as the implicit baseline.

Each stem completion trial was followed by a forced‐choice recognition task in which participants were presented with two possible words of the same three‐letter stem and a third option to say “another word.” Participants were asked to select the option corresponding best to the word they had recollected in the preceding stem completion trial or to select the option corresponding best to the word they thought was studied at encoding if they had failed to recollect it. Although one of the two presented words in this forced‐choice task was always the studied word, participants were not told this so that, if they failed to recognize the studied word from the list of two words, they would not guess which of the two was correct but select the “another word” option. Each forced‐choice trial lasted for 3.5 s preceded by a central fixation cross presented for 800 ms.

Again, responses in this task were collected using a special MR‐compatible response box and participants used both hands to choose one of the five response choices (R1, R2, R3, Ra, and N) in the stem completion task. Three fingers from one hand were used for the recollection rating, while two fingers of the other hand were used for the extra two responses. The allocation of the recollections responses to the left or the right hand was counterbalanced across participants. In the forced‐choice recognition trials, participants used three buttons to select the first or the second word or to select “another word.” Two fingers from one hand were used for the word selection options, while one finger of the other hand was used for selecting “another word.” The allocation of these options to the left or the right hand was counterbalanced across participants. In total, the three encoding‐test cued recall cycles lasted for 1,534.5 s (i.e., 25.57 min). Only data from the test block are presented in the Results.

### 2.4 fMRI acquisition and data analyses

Scanning was conducted using a 3T Philips Achieva scanner (Philips Healthcare, Eindhoven, The Netherlands) with an eight‐element SENSE head coil. A dual echo‐planar imaging (EPI) sequence covering the whole brain was implemented with a long TE (echo time) of 35 ms and a short TE of 12 ms. Multi‐echo fMRI has previously been shown to reduce image distortion in areas susceptible to signal loss (Poser & Norris, 2009) and it has recently been used in the anterior temporal area (Halai, Welbourne, Embleton, & Parkes, 2014). A total of 528 volumes were collected from each individual with 42 slices per volume, TR = 3.7 s and 2.5 mm × 2.5 mm × 3 mm voxel size (no gap; 96 × 96 acquisition matrix). A T_1_‐weighted high‐resolution structural image was also collected for each participant for anatomical reference and coregistration with a 1 mm isotropic voxel size (180 slices; matrix size 256 × 256). Soft pads were used during the MRI session to stabilize each participant's head and noise cancelling headphones and earplugs were provided.

Simple linear summation was used to combine the images produced by the two echo times for each TR and image volume. These data were then analyzed using Statistical Parametric Mapping (SPM8) software (Wellcome Trust Centre for Neuroimaging, London, United Kingdom, http://www.fil.ion.ucl.ac.uk/spm/). The functional data were realigned to the first image using a six‐parameter rigid body transformation, resliced using sinc interpolation and slice‐time corrected to the middle slice to account for differences in slice acquisition times. The T_1_ images from each participant were coregistered to the corresponding mean EPI image. Spatial normalization to the Montreal Neurological Institute (MNI) template was performed using DARTEL as implemented in SPM8 (Ashburner, 2007). Finally, an isotropic 8 mm full‐width half maximum (FWHM) Gaussian smoothing kernel was applied to the functional images.^1^

For each participant, the response outcomes from the cued recall test and familiarity test tasks were analyzed at the subject level using the general linear model (GLM) implementing a canonical hemodynamic response function convolved with a series of delta functions corresponding to the onset of each event. The conditions of interest for the cued recall task included R1, R2, R3, and Ra responses as well as misses (M) and false recollections (FR). FR trials included the events for which participants provided a recollection rating (R1, R2, or R3) but failed to select the correct word in the subsequent forced‐choice trial. M trials included stems of studied words which participants indicated did not begin any of the studied words, that is, the stems were new. Regressors of no interest included trials with no behavioral response. Additionally, the six movement parameters produced at realignment were also included in the model to capture residual movement‐related effects. The model for the familiarity task for each subject included the familiarity responses (F1, F2, and F3) along with correct rejections (CR), misses, and inadvertent recollections (R). Trials with no behavioral response and the six movement parameters from realignment were also included in this model.

#### 2.4.1 Parametric analyses

For each participant, activity modulations by recollection and familiarity were analyzed using separate parametric models for the reported recollection (R1, R2, and R3) and familiarity strength (F1, F2, and F3). Specifically, recollection hits (in the cued recall task) and familiarity hits (in the familiarity‐only task) were entered as conditions of interest, and the reported strength accompanying these decisions was convolved with the stimulus‐related Hemodynamic Response Function (HRF; Büchel, Holmes, Rees, & Friston, 1998). In both models, the recollection and the familiarity one, misses were also used as the level reflecting zero recollection (R_0_) and zero familiarity (F_0_), respectively. Both activation and deactivation patterns with increased recollection and familiarity strength were examined, at the subject level, using parametric *t* contrasts. Linear effects were modeled, but the nonlinear quadratic effects were also included in the model to capture variance in each individual model that was not explained by the linear effects. At the group level, the quadratic contrasts (for both the recollection and the familiarity parametric effects) did not produce any major unique activations that were not also captured by the linear contrasts and therefore are not reported separately.

Using a similar methodology, three additional parametric models, capturing different degrees of change of strength and amount, running from most change of strength to most of amount, respectively, were compared in the cued recall task to explore the extent to which the hippocampus responds predominantly to recollection strength or amount recalled. The *strength* model included R1, R2, and R3 responses. In this model, the amount of information recalled was constant (i.e., the cued word) while the strength was variable from weak to strong. A mixed strength/amount model included M, weak recollection (R_weak_), and strong recollection (R3) responses, where R_weak_ responses comprised the collapsed R1 and R2 responses. This model involves one increment in amount recalled from M (no recall) to R_weak_ (weak cued recall of the word), but no difference in the amount recalled between R_weak_ and R3. Finally, the *amount* model incorporated stepwise increments in the amount of information that is recalled and included the following types of responses: M (no recall), R3 (cued recall of the word), and Ra (cued recall of word plus additional recollection). Parameter estimates were extracted for each subject from a hippocampal region using a 6 mm sphere centered around an area identified in two of the parametric analyses (MNI xyz: −18 −7 −14). A series of curve estimation regression analysis were then conducted for each participant to examine the degree of the relationship between the hippocampal activity and the response to the three models (strength versus amount). Specifically, the β‐coefficients showing the slope of the relationship were extracted for each participant and each model (i.e., three βs per subject) and were then compared at the group level using a one‐way analysis of variance (ANOVA) and Bonferroni corrected pairwise comparisons.

Direct *t* contrasts comparing Ra with R3 or Ra with all the other levels of reported recollection strength collapsed (R1, R2, and R3) were also run. All contrasts involving Ra responses were run on a subset of participants (*n* = 9) with enough Ra trials (at least nine trials). The parametric and categorical contrasts at the subject level were used to analyze the effects at the group level—treating participants as a random effect—using one‐sample *t* tests. The direct contrasts among cued recall (R3), additional recollection (Ra), and familiarity (F3) were set up as second‐level paired *t* tests in SPM. The produced SPM maps were thresholded at an uncorrected level of *p* < .001. Whole‐brain activations are reported as significant if they survived a cluster‐wise family‐wise error (FWE)‐correction of *p* < .05 (using Random Field Theory). Due to our a priori hypothesis, hippocampal activations are small volume FWE‐corrected at *p* < .05 (SVC) for the entire bilateral hippocampus (anatomical mask from WFU PickAtlas toolbox). For consistency and to enable comparison with previously published work, where appropriate, activations not surviving the FWE‐correction level are also reported (or denoted in the tables) at the uncorrected *p* < .001 level. Finally, activation data (parameter estimates) from the entire cluster of activity are plotted in the figures.

#### 2.4.2 Behavioral analyses

Memory accuracy [hit rate/(hit rate + FA rate)] and reaction times (RT) in both tasks were analyzed using one‐way anovas with recollection (R1, R2, and R3) or familiarity (F1, F2, and F3) strength as the between‐subjects factor. Paired *t* tests were also used to directly compare the accuracy and RT between Ra responses and the other levels of reported recollection strength. A conventional significance level of *p* < .05 was adopted for all behavioral analyses.

## 3 RESULTS

### 3.1 Cued recall task

#### 3.1.1 Behavioral data

Word cued recall/recollection accuracy for each of the three levels of recollection (R1: M = 0.73, *SD* = 0.26; R2: M = 0.81, *SD* = 0.16; R3: M = 0.91, *SD* = 0.08) and the additional recollection option (Ra: M = 0.94, *SD* = 0.12) was significantly higher than chance (R1: *t*(16) = 3.67, *p* = .002; R2: *t*(16) = 7.79, *p* < .001; R3: *t*(16) = 20.36, *p* < .001; Ra: *t*(10) = 11.87, *p* < .001). The one‐way ANOVA across the three levels of recollection showed a significant linear increase in recollection accuracy with increased recollection strength (R3 > R2 > R1; *F*(2, 32) = 7.44, *p* = .002, η^2^ = 0.32). Importantly, R3 and Ra accuracy were closely matched, *t*(10) = −0.91, *p* = .39). The one‐way ANOVA on the RT data showed a main effect of response (*F*(2, 32) = 3.71, *p* = .04, η^2^ = 0.19) indicating shorter response latencies with increasing levels of reported recollection strength (R1 > R2 > R3). Finally, RTs to Ra responses (M = 2,175 ms, *SD* = 449 ms) were not significantly different than R1 and R2 (R1: M = 2045 ms, *SD* = 318 ms; R2: M = 1994 ms, *SD* = 378 ms). However, there was a trend (*t*(10) = −2.16, *p =* .056) for R3 responses (M = 1826 ms, *SD* = 348 ms) to be faster than Ra responses (M = 2,176 ms, *SD* = 449 ms).

#### 3.1.2 Imaging data

**Figure 2:**
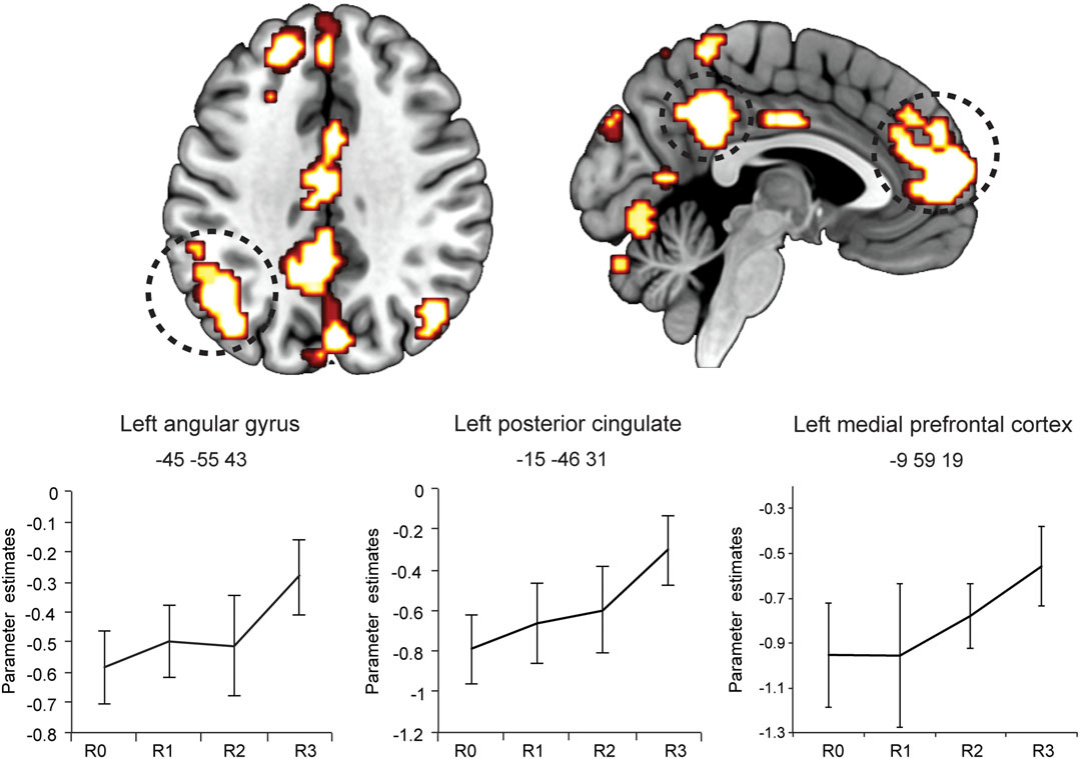
Brain regions responding to recollection strength at retrieval and parameter estimate plots. Error bars show the *SEM*. R0 = recollection misses; R1 = weak recollection; R2 = moderate recollection, R3 = strong recollection [Color figure can be viewed at wileyonlinelibrary.com]

The brain regions that were identified as responding to the strength of the reported recollection are presented in Table 1 and Figure 2. Only areas showing increased activity to increasing levels of reported recollection strength were identified, whereas no brain region was found to decrease its activity with increasing reported levels of recollection. As shown in Figure 2, the brain regions responding to recollection strength formed an extensive network of brain regions including the left superior medial (BA 10), superior lateral (BA 8/9), and inferior (BA 45) prefrontal cortex, the bilateral angular gyrus (BA 39/40), the bilateral precuneus (BA 7), the left posterior cingulate cortex (including the retrosplenial cortex; BA 31), and the bilateral middle temporal gyrus (BA 21). Importantly, the hippocampus was not found to respond to recollection strength (either by increasing or decreasing its activity) even at a considerably lower threshold (*p* = .01, uncorrected).

**Table 1:** Parametric increases in activity across the four levels of reported recollection (R_0_–R3)

Side	Region	No. of voxels	~BA	MNI x y z	*t* value
L	Medial frontal gyrus	593	BA 10	−9 59 19	9.78
L	Superior frontal gyrus		BA 8/9	−15 41 34	5.86
L	Middle frontal gyrus	148	BA 8	−21 17 43	6.83
L	Angular gyrus	279	BA 39/40	−45 −55 43	6.59
R	Angular gyrus	121	BA 39/40	45 −61 52	5.96
R	Cerebellum	132		33 −73 −35	5.85
R	Precuneus	76	BA 7	3 −70 31	5.81
L	Precuneus	62	BA 5/7	0 −46 67	4.64*
L	Posterior cingulate cortex	202	BA 31	−15 −46 31	5.72
R	Lingual gyrus	92	BA 18	21 −70 −5	5.12
R	Middle cingulate gyrus	67	BA 24	3 5 34	4.8
L	Insula	44	BA 13	−39 8 −8	5.2*
L	Inferior frontal gyrus	23	BA 45	−48 41 −14	5.16*
R	Middle temporal gyrus	16	BA 21	69 −25 −2	4.8*
L	Middle temporal gyrus	12	BA 21	−66 −40 −5	4.24*

*Note*: *p < .001, uncorrected. All the other effects are FWE‐corrected at the cluster level.

#### 3.1.3 The hippocampus responds to amount of recalled information

**Figure 3:**
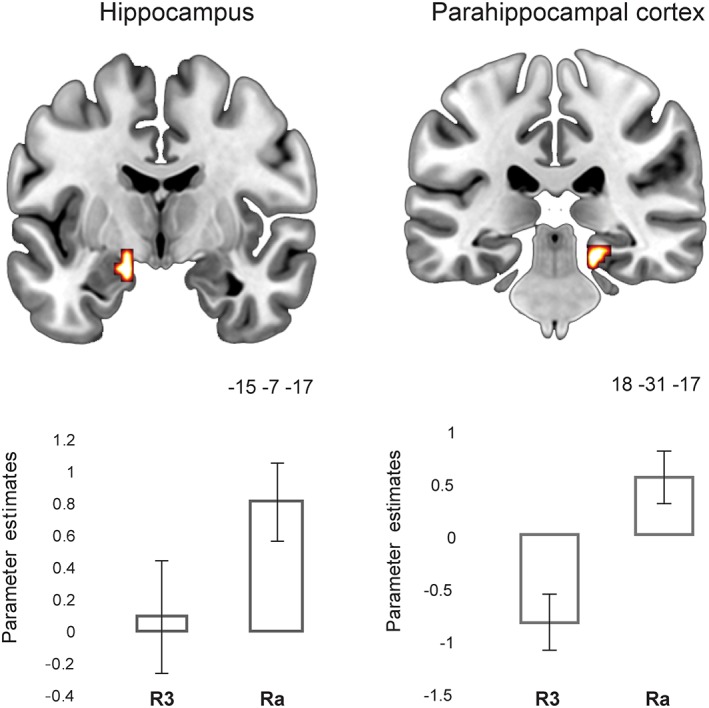
Medial temporal lobe responses at retrieval, when comparing additional recollection versus strong recollection, Ra > R3. Error bars show the *SEM*. Ra = additional recollection; R3 = strong recollection. Parameter estimates within these regions for other conditions within the cued recall task are plotted in Supporting Information Figure S1 [Color figure can be viewed at wileyonlinelibrary.com]

Despite the lack of activity modulation in the hippocampus for recollection strength, additional recollection (Ra) resulted in greater hippocampal activation when contrasted to levels of recollection strength. Specifically, two clusters within the bilateral hippocampus (left: −15 −7 −20, SVC FWE‐corrected, *T* = 5.43; Right: 21 −25 −11, SVC FWE‐corrected, *T* = 5.23) were found to be more active for Ra than the collapsed R strength (R1, R2, and R3) responses. Importantly, when comparing Ra to R3 responses, which are characterized by matched accuracy levels (see Behavioral data 3.1.1), a cluster within the left anterior hippocampus (−15 −7 −17; SVC FWE‐corrected, *T* = 7.19) was found to respond to Ra versus R3 responses (Figure 3). Even when we controlled for the trend in RTs to be faster for R3 versus Ra (by including RTs in the GLM), the same pattern of activity was observed in the hippocampus. Other extra‐hippocampal areas that responded more to Ra relative to R3 (see Supporting Information Table S1) included the right PHC (BA 35; Figure 3), the left angular gyrus (BA 39), the right precuneus (BA 7), the left middle temporal gyrus (BA 21), the left middle frontal gyrus (BA 8), and the left middle occipital gyrus (BA 19). The opposite contrasts (R1, R2, and R3 combined > Ra and R3 > Ra) did not result in any significant activation. As these effects that were related to Ra responses were based on a smaller sample of participants with enough Ra trials (*n* = 9), the individual participant activations within the hippocampus (using an anatomical mask) were also inspected for the critical contrasts (i.e., Ra > R3 and Ra > R collapsed strength). This analysis (Supporting Information Table S3) indicated significant activations within the hippocampus for each one of the nine participants for both contrasts involving Ra responses. Therefore, the group effect indicates reliable activation of the hippocampus when additional recollection is involved.

These findings stress the sensitivity of the hippocampus to the amount of information that is recalled. To further explore this effect, three additional parametric models were tested with the aim of investigating whether the hippocampus responds more to the subjective strength of recollection or the amount of information recalled. These models included three levels, but varied with respect to their emphasis either on changes in recollection strength or amount recalled (see section 2).

The *strength* model (R1, R2, and R3 responses) was characterized by constant amount of information recalled but variable memory strength. The *mixed* strength/amount model (M, R_weak_, and R3) was characterized by a single increment of the amount recalled from M to R_weak_, but no difference in the amount recalled between R_weak_ and R3. Finally, the *amount* model incorporated stepwise increments in the amount of information that is recalled and included M, R3, and Ra levels. In this model, R3 and Ra responses, as noted above, are characterized by matched accuracy/strength and, therefore, are considered to predominantly capture amount variations (i.e., stepwise increments in recollection).

**Figure 4:**
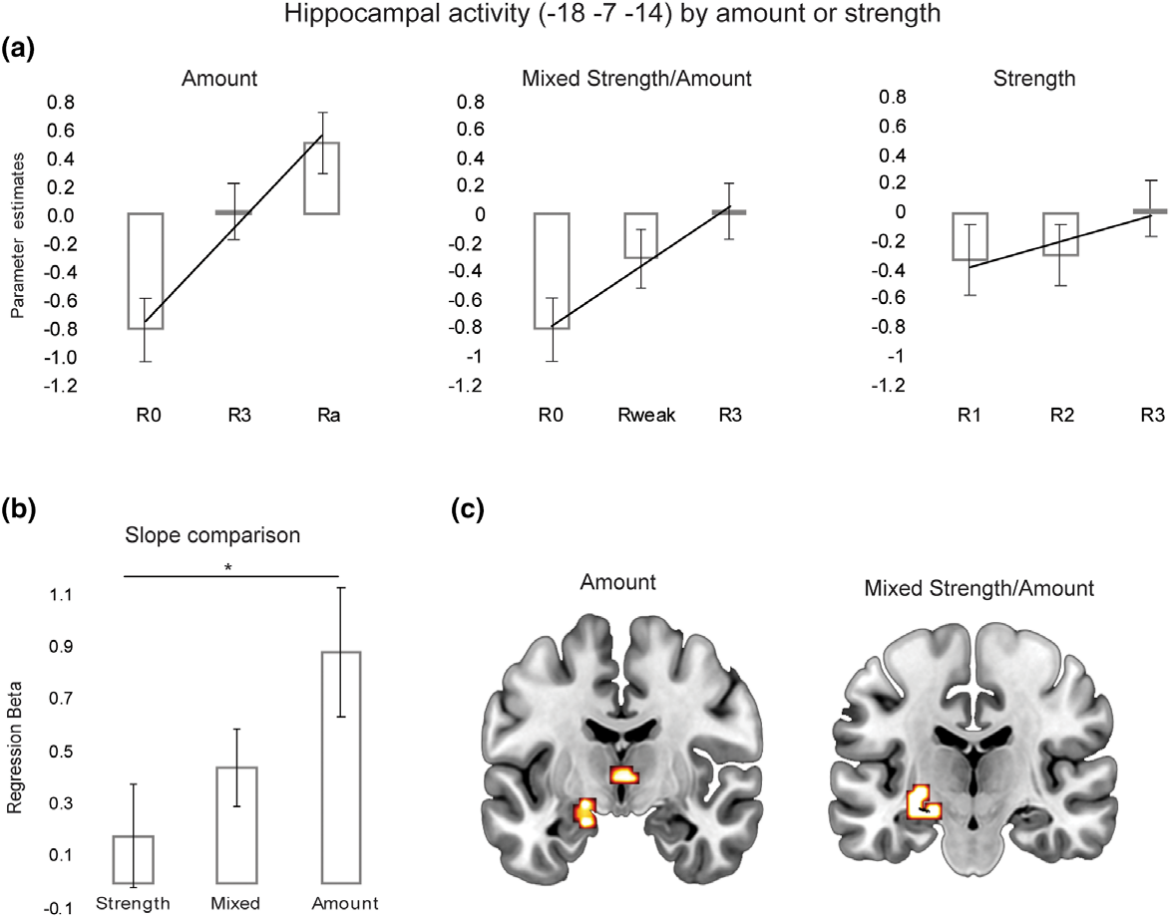
Comparison of hippocampal activations at retrieval as a response to strength and amount of recollection. (a) Parametric hippocampal activations in three GLMs modeling amount and strength of recollection; (b) regression analysis β‐coefficients comparison between the strength, the mixed strength/amount and the amount models; and (c) activations within the hippocampus (−18 −7 −14) found for the amount and the mixed strength/amount parametric models. R0 = recollection misses; R_weak_ = R1 + R2 (collapsed); R3 = strong recollection; Ra = additional recollection. *p < .05 [Color figure can be viewed at wileyonlinelibrary.com]

Consistent with the first parametric analysis with recollection strength reported above, the strength model did not result in any hippocampal activation even at a very low threshold (*p* = .01, uncorrected). On the other hand, as shown in Figure 4c, one cluster within the left anterior hippocampus (−18 −7 −14; SVC FWE‐corrected, *T* = 4.85) was identified in the mixed strength/amount model as increasing its activity across the three levels. The same area was also found to be active in the case of the amount model (SVC FWE‐corrected, *T* = 4.95) along with another left hippocampal area within the same cluster (−15 −10 −20). Finally, another analysis was carried out to help determine how much *strength* and *amount* sensitivity contributed to activation in the anterior hippocampal area (−18 −7 −14). A curve estimation regression analysis was performed for each participant on the extracted parameter estimates from this area and the three levels of the three models (Figure 4a,b). The slopes of the relationships between strength or amount, respectively, and activity in the anterior hippocampal cluster (6 mm sphere centered at −18 −7 −14) were compared across the nine subjects with sufficient Ra responses. The β‐coefficients were significantly different across the three models (*F*(2, 16) = 7.29, *p* = .006, η^2^ = 0.48) indicating a significant linear increase (*F*(1, 8) = 9.85, *p* = .014, η^2^ = 0.55) of the β‐coefficients as recollection amount loading increased (strength model < mixed model < amount model). This means that the slope of the relationship is stronger (steeper) in the anterior hippocampus when there is a heavier loading on the amount modulation than the strength modulation.

### 3.2 Familiarity task

#### 3.2.1 Behavioral data

In the familiarity task, familiarity accuracy across the three levels of reported F was significantly above chance levels of performance (F2: *t*(16) = 8.61, *p* < .001; F3: *t*(16) = 32.24, *p* < .001) with the exception of F1 (*t* < 1), which reflects the weaker memory level of familiarity that is reported by the participants. As was the case in the recollection task, RTs were shorter with increasing levels of familiarity strength (*F*(2, 32) = 34.08, *p* < .001; F1 > F2 > F3, all *p*s < .002).

#### 3.2.2 Imaging data

**Figure 5:**
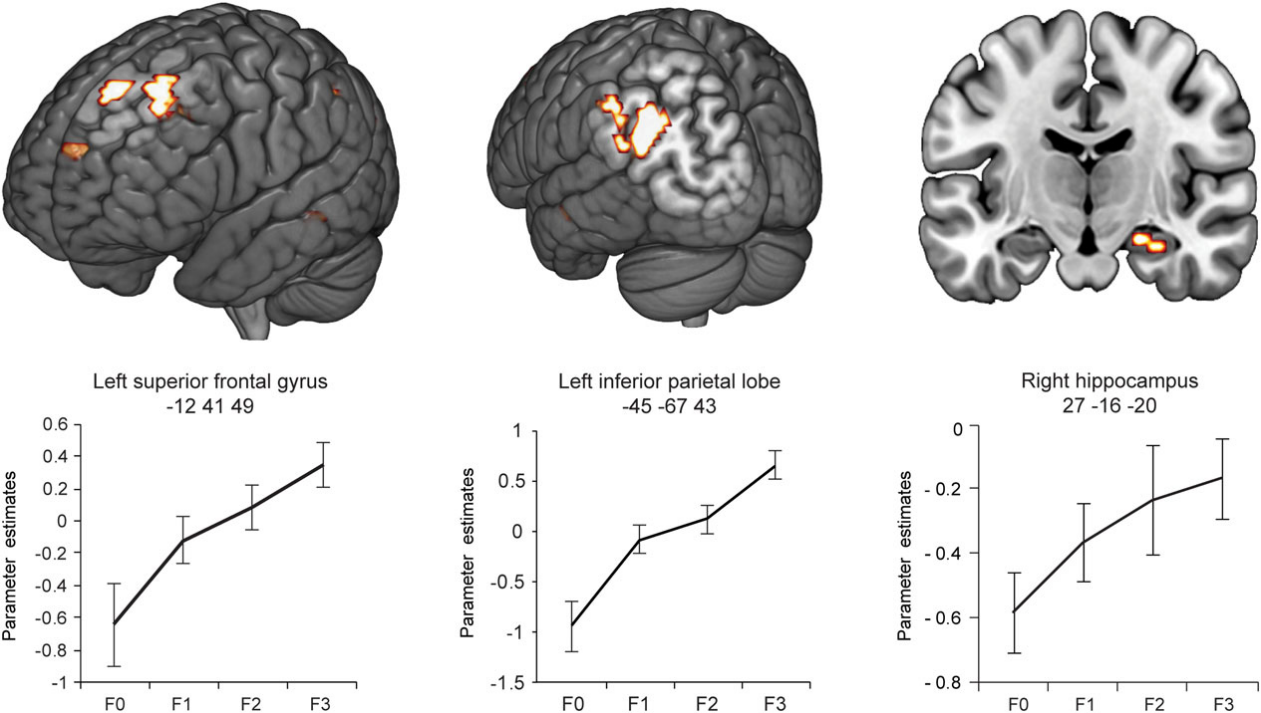
Brain regions increasing their activity at test as a function of word familiarity strength. Error bars show the *SEM*. F0 = familiarity misses; F1 = weak familiarity; F2 = moderate familiarity; F3 = strong familiarity [Color figure can be viewed at wileyonlinelibrary.com]

The familiarity network (Table 2 and Figure 5) included the brain regions that increased (or decreased) their activity across the four levels of familiarity strength (F0, F1, F2, and F3). Activity increases with increases in reported familiarity were found in areas within the left inferior parietal lobe (BA 39/40), the left middle and superior frontal gyrus (BA 8/9), and one cluster in the left precuneus (BA 7). Within the MTL, a cluster in the right hippocampus (27 −16 −20; Table 2 and Figure 5) was also found to respond to the reported familiarity of the word stimuli (at *p* < .001, uncorrected; extent = 9 voxels). No other effects were isolated in the MTL and no region was found showing decreased activity with increases in familiarity strength.

**Table 2:** Parametric increases in activity across the four levels of reported familiarity (F_0_–F3)

Side	Region	No. of voxels	~BA	MNI x y z	*t* value
L	Inferior parietal lobe /angular gyrus	166	BA 39/40	−45 −67 43	7.2
L	Middle frontal gyrus	42	BA 8/9	−36 32 40	5.57
L	Superior frontal gyrus	28	BA 8	−12 41 49	5.42*
L	Precuneus	10	BA 7	−12 −55 28	4.44*
R	Hippocampus	9		27 −16 −20	4.02*

*Note*: *p < .001, uncorrected. All the other effects are FWE‐corrected at the cluster level.

### 3.3 Comparisons among cued recall, additional recollection, and familiarity

A direct comparison between strong cued recall (R3) and strong familiarity (F3) was also conducted (both R3 > F3 and F3 > R3; see Supporting Information Table S2). For R3 > F3, regions that survived correction for multiple comparisons included the bilateral middle occipital gyrus (BA19), the left inferior parietal lobe (BA40), the superior frontal gyrus (BA6), the right superior parietal lobe (BA7), the left inferior frontal gyrus (BA45), and the ventral lateral thalamus. For F3 > R3, areas of activity included the caudate nucleus, the bilateral angular gyrus (BA39/40), the left superior frontal gyrus (BA8/10), and the left precuneus (BA7).

**Figure 6:**
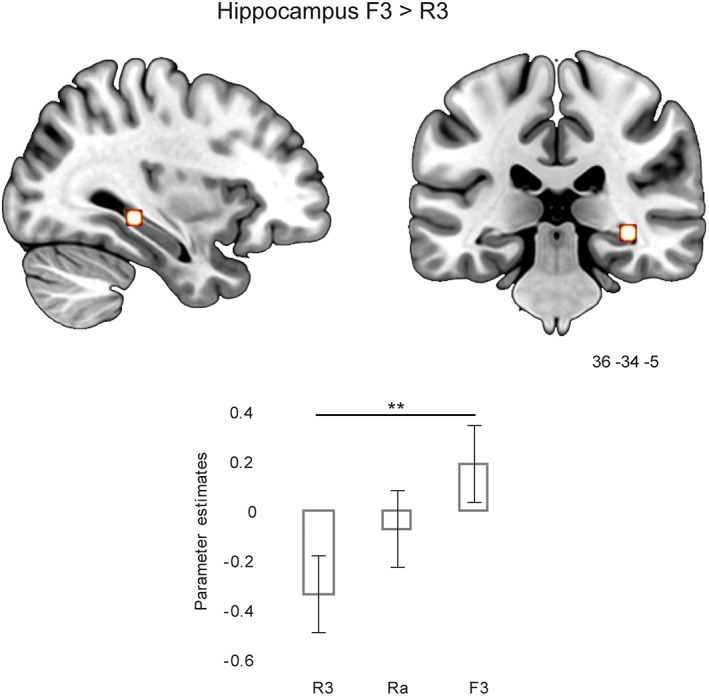
Hippocampal activation at test when comparing strong familiarity (F3) and strong recollection (R3) for word stimuli and comparison of parameter estimates for R3, Ra, and F3. **p < .01. Parameter estimates within this region for the other conditions in the cued recall and familiarity tasks are plotted in Supporting Information Figure S1 [Color figure can be viewed at wileyonlinelibrary.com]

Importantly, in the MTL, a hippocampal cluster in the right posterior hippocampus (36 −34 −5; 10 contiguous voxels at *p* < .001, uncorrected) was more active for F3 than R3 responses. Finally, contrasting Ra versus F3 (Ra > F3) did not give any significant activation in the MTL (including the hippocampus), instead areas such as the left fusiform gyrus (BA19/37), the left middle occipital gyrus (BA19), and the right middle temporal gyrus (BA37) were more active for Ra than F3 (see Supporting Information Table S2). The opposite contrast F3 > Ra did not produce any significant effects. This finding indicates that the selective hippocampal activation to familiarity (both in the parametric model and in the F3 > R3 contrast) is of the same or equivalent magnitude as the hippocampal activation for Ra. To further qualify this, the parameters estimates within the hippocampal region identified in F3 > R3 (36–34 ‐5) were compared among F3, R3, and Ra. Consistent with the lack of hippocampal activation for Ra > F3, the parameter estimates were significantly different between F3 and R3 (*t*(16) = 3.57, *p* = .003) but no significant difference between F3 and Ra was found (*t* < 1; see Figure 6).

## 4 DISCUSSION

### 4.1 The hippocampus is sensitive to amount recalled not to recollection strength

Our study found two clear effects relating recollection‐like memory to hippocampal activity. First, even when word‐related recall accuracy and confidence remained constant, as amount recollected increased, hippocampal activity increased. Second and in contrast, when the amount of verbal material recalled remained constant, increases in confidence and accuracy had no effect on hippocampal activity. These findings strongly suggest that, at least for words, the hippocampus is sensitive to increases in the amount of information in recall memory, but not to confidence in recall or recall accuracy, as the study of Qin, van Marle, Hermans, and Fernandez (2011) also suggested for scenes.

Several brief comments on these findings about the sensitivity of the hippocampus to these features of cued word recall are warranted. The dissociation between amount of information recalled and strength was made possible by the novel cued recall procedure in which participants often did not report that they had recalled additional information related to the earlier encoding of a studied word, recalling only that word at one of three levels of confidence. These findings strongly suggest that the hippocampus is insensitive to cued recall strength on its own.

Accuracy variability shown by word cued recall was not high because false alarm levels remained fairly low even with low confidence levels. However, although the amount of accuracy change did not modulate hippocampal activity significantly, it was sufficient to modulate activity in other brain structures, including the parahippocampal, retrosplenial, medial frontal, and inferior parietal cortices. At the very least, the hippocampus is likely to be less sensitive to word cued recall strength changes than these structures. This conclusion is also consistent with Qin et al.'s (2011) similar findings with scenes, using a very different method. Interestingly, this hippocampal insensitivity to word cued recall strength/accuracy/confidence is similar to its insensitivity to familiarity strength/accuracy/confidence for objects, scenes, and new faces (see Kafkas et al., 2017), although, in both cases, other brain structures are sensitive.

When the word cue only led to recall of a studied word, there was less activation of the hippocampus than when additional information was recollected, but even cued recall of a word stimulus alone was probably sufficient to activate the hippocampus significantly. Although such activation was hard to detect, the parametric analysis of failed recall (M), word recall alone (R3), and additional recall (Ra; i.e., the amount model) was significant even though power was reduced because fewer participants could be included in this analysis. Specifically, the significant parametric activation produced by M, R3, and Ra is driven as much by the change from M to R3 as it is from R3 to Ra (see Figure 4). In contrast, in the mixed strength/amount model, the hippocampal activity is driven by the change from M to R_weak_, but not by the change from R_weak_ to R3 as indicated in the strength model. This means that the hippocampus responds in an increasing function when we move from failed recall (M) to recall for the word alone (R3) and its activity increases further when comparing R3 to additional recall (Ra). This finding suggests that, although a word stem usually led to the recall of a studied word alone, there was sufficient information in memory to activate the hippocampus. Qin et al. (2011) drew a similar conclusion about verbally cued recall of scene gist. This structure, therefore, must be sensitive to cued recall even when minimal amounts of information are recalled.

### 4.2 Hippocampus and familiarity memory

As indicated in section 1, some researchers (e.g., Smith et al., 2011) argue that hippocampal activity is increased by increases in familiarity and recollection strength/accuracy in the same kind of way. Apparently consistent with this claim, Smith and colleagues reported that equally strong word familiarity and recollection activated the hippocampus equally and more than weak familiarity and presumably recollection. Although we used a different procedure for measuring increases in familiarity strength from Smith et al. (2011), like them, we also seemed to find that hippocampal activity increased as word familiarity accuracy increased. It should be noted here that this activation did not survive strict FWE‐correction. However, given the long debate regarding the proposed role of the hippocampus in familiarity memory and the fact that previous research on the topic conventionally used a reduced cluster‐wise threshold for the MTL at an uncorrected level of inference (e.g., Smith et al., 2011), this finding is given further discussion.

At first sight, our findings, like those of Smith and colleagues, seem to suggest that the hippocampus is sensitive to familiarity accuracy increases. If correct, this first interpretation, given our previous results with visual stimuli, such as scenes, objects and faces (Kafkas et al., 2017; Kafkas & Montaldi, 2012; Montaldi et al., 2006), implies that words differ from these kinds of visual stimuli. Most pictures of scenes, objects, and new faces are relatively novel prior to study but this cannot be true of words that have been encountered thousands of times previously. Experimental recognition tests are, either explicitly or by implication, asking participants to identify stimuli that were encountered in a designated study context rather than as just having been encountered before somewhere on one or more occasions. Study of previously very low familiarity (or completely novel) pictorial stimuli is very likely to make them clearly more familiar. It can, therefore, confidently be inferred that they have been encountered in the study context accurately when it feels that they have ever been encountered before. In other words, poststudy item familiarity can be sufficient for recognition of these visual stimuli without the need for a memory relating the stimulus to a specific study context.

In contrast, as all words must be familiar from myriad previous encounters, it is much less clear that a study session will raise word familiarity enough to make an accurate recognition judgment. Another possibility, suggested by Smith et al. (2014), is that word recognition is supported by word‐context associative familiarity, which, like recollection, is supported by the hippocampus. As yet, there is no evidence that this form of associative familiarity exists and Mayes et al. (2007) argued that it will not unless the relevant word and context information converge in the neocortical regions of the MTL through a PRC–PHC interaction, in which case the associative familiarity will hippocampal independent.

No evidence yet bears directly on these contrary views of word‐context familiarity. Thus, although Bird and Burgess (2008) concluded from a meta‐analysis that relatively selective hippocampal lesions disrupt word recognition but not face recognition, they did not clearly distinguish between whether hippocampal lesions disrupt any form of word familiarity, including word‐context familiarity, or whether any form of familiarity supports word recognition fairly poorly. The latter possibility would imply that recognition must depend mainly on recollection of diagnostic details from the study episode, which is dependent on the hippocampus. The effect of hippocampal lesions on any form of word familiarity is, therefore, currently unresolved by available literature (see Montaldi & Mayes, 2010).

Several aspects of our results are inconsistent with previous views (e.g., Smith et al., 2011) about the relationship between word familiarity and the hippocampus. Our results show a linear relationship between word familiarity strength/accuracy and hippocampal activity. In contrast, proponents of the view that the hippocampus mediates word familiarity (e.g., Smith et al., 2011; Song, Jeneson, & Squire, 2011; Squire et al., 2007) have argued that increases in recognition strength/accuracy affect hippocampal activity in a nonlinear way. They argued that any increase in hippocampal activity will be so small as to be undetectable until familiarity is very confident and strong/accurate, but that is not what our familiarity parametric results showed. More importantly, we also found that increases in word cued recall, which matched amount of information in memory with word item familiarity, did *not* increase hippocampal activity as its strength increased. Assuming that information in familiarity memory cannot by definition increase as its strength/accuracy increases, then familiarity should behave like cued recall, according to Smith et al. (2011) and Squire et al. (2007), for example, but our findings show that it does not.

This tension suggests another interpretation of why our present familiarity findings with words and previous visual stimulus findings (Kafkas et al., 2017; Kafkas & Montaldi, 2012; Montaldi et al., 2006) are discrepant with respect to hippocampal activity. Measuring familiarity for words with current versions of the remember/know procedure may be seriously confounded with unreported and undetected recollection, which increases as confidence and accuracy of recognition memory increase. The problem is likely to apply equally to Smith et al.'s (2011) and our different methods of assessing word familiarity. If so, the amount of recalled information in “familiarity memory” would increase as confidence and strength/accuracy increased in both experiments. This recall relates exclusively to when words are used as recognition cues. Myriad previous encounters ensures that words are never encoded, re‐encoded, or retrieved as a series of letters but always in relation to a meaning, close associates, or meaning‐related visual imagery. This recall imposes a challenge when recognition memory is probed using the remember/know procedure as this recollection‐like activity is more likely to be deemed a feeling of familiarity (or a “know” response) due to how words, meaning, close associates, and imagery are inextricably connected.

Specifically, in fMRI studies of recognition memory, stimuli are encoded and then re‐encoded and recollection involves the cued recall of any memory information associated with the earlier encoding of the stimulus. What we argue here is that stimulus encoding will vary across occasions and the boundary between re‐encoding a stimulus and recalling associated details of it on the previous encoding is not completely clear‐cut but depends on the type of stimulus and the type of the encoding task. In the previous fMRI studies with pictorial stimuli (Kafkas et al., 2017; Kafkas & Montaldi, 2012, 2014; Montaldi et al., 2006), our low‐level encoding conditions for scenes, objects, and faces have encouraged the encoding of visual features while our familiarity‐only instructions have made it even more unlikely that associated semantic details would be retrieved. Even if they were, this would be clearly construed as recalling one's thoughts about the stimulus from encoding rather than as stimulus identifying re‐encoding.

In contrast, both the present word fMRI study and previous ones (e.g., Smith et al., 2011) required participants to make semantic judgments. Particularly, as noted above, encoding of visually presented words, which are visual symbols with many semantic associations (denotations, connotations, and looser associates), may have led people to focus attention more on the symbols' semantics and associated visual imagery rather than their visual features (i.e., the presented letters). At test, it seems likely that participants would have recalled some of the associates that they encoded at study. However, as these word‐related associates are inextricably linked to the words themselves, this may have felt to participants as if they were merely re‐encoding the study word rather than recollecting associated context features from earlier study and hence wrongly reported as familiarity.

As our results also showed that increasing information in recollection/recall memory increases hippocampal activity even when strength/accuracy does not change, this interpretation suggests that, with greater accuracy/strength, familiarity would activate the hippocampus more than even the most confident cued recall where there would be less information in memory. This is exactly what we found. In contrast, hippocampal activity did not differ between additional recollection and equally strong/accurate “familiarity.” Such findings are what would be expected if, despite extensive training (see Migo et al., 2012), participants increasingly failed to detect and report recollection as their confidence in their familiarity judgments increased. Provided that participants often failed to detect and report recollection, we would expect that they would fail to report recollection of greater amounts of information as their “familiarity” confidence increased because recollection is very diagnostic of recognition. Therefore, this point provides preliminary evidence suggesting that familiarity responses to word stimuli were contaminated by recollection that contained increased amounts of information the more confident participants became.

Another inconsistency with previous familiarity effects with pictorial stimuli was that unlike confidence/accuracy in familiarity for scenes, objects, and faces (Kafkas et al., 2017), confidence/accuracy in “familiarity” for words did not influence the PRC, PHC, or entorhinal cortex even when statistical thresholds were lowered. Nor, unlike familiarity for scenes, objects, and faces, did it influence activity in the dorsomedial nucleus of the thalamus (Kafkas & Montaldi, 2012). A few brain regions, such as the neocortical MTL structures and parts of the thalamus, seem to be selectively sensitive to familiarity (see, e.g., Edelstyn, Mayes, & Ellis, 2014; Kafkas & Montaldi, 2012, 2014), but in the present study these regions were insensitive to word “familiarity.” Although these observations are based on null findings (i.e., not finding expected effects), these findings seem to be more consistent with our interpretation of our hippocampal “familiarity” results, which suggest that participants were failing to report that they were recollecting increasing amounts of semantic information about the words as “familiarity” confidence increased.

### 4.3 LIMITATIONS, IMPLICATIONS, AND CONCLUSIONS

One limitation of the present study is the small number of participants, especially in the analyses involving Ra responses. Although an individual subject analysis (Supporting Information Table S3) showed that the critical hippocampal activations in the contrasts involving Ra are reliable for each participant, it will be valuable for future research to replicate this finding using a larger group of participants. Also, in this study, we performed a whole‐brain analysis and therefore DARTEL normalization was deemed more appropriate. However, as the critical findings reported in the article involved activations within the MTL, further optimization protocols (e.g., MTL [ROI] realignment techniques as in Yassa & Stark, 2009) may be adopted in future studies. Finally, the reader should also note the greater contextual overlap between study and test characterizing the cued recall task (both inside the scanner) than the familiarity task (study outside the scanner versus test inside the scanner) when considering direct comparisons between the two tasks as reported in section 3. This was dictated by restrictions in the amount of time participants were allowed to stay in the scanner due to ethical considerations and facility policies.

Our results have three other general implications. First, future fMRI comparisons of the effects of stimulus familiarity and recollection on the hippocampus should focus on matching how much memory information each contains. Matching recognition strength (operationally defined as accuracy; see, e.g., Squire et al., 2007) appears to be unimportant as we and Qin et al. (2011) have shown. In contrast, the matching amount of memory information is needed to determine whether familiarity does not activate the hippocampus as by definition item familiarity holds less memory information than recollection.

A further desideratum for answering this question is to what extent the measure of familiarity can be trusted in the case of word stimuli. Before determining whether hippocampal involvement with familiarity for words is an exception relative to other visual stimuli, remember/know instruction procedures need to be further modified to try and avoid the ambiguity we have noted above. Future work should also vary encoding and test instructions, differentially stressing semantic or sensory stimulus features, to determine whether participants' increasing focus on semantics leads to greater misreporting of recollection as familiarity.

Finally, our results suggest that the conceptual dividing line between stimulus familiarity and recollection is more blurred than has been previously supposed. As semantic information needs to be recalled whereas perceptual does not necessarily, it could be that the contrast of semantic to perceptual information is as important for the hippocampus as the familiarity/recollection contrast.

## Supporting information

Supporting Information

## Footnotes

1 A lower smoothing kernel (5 mm isotropic) did not result in marked changes to the critical findings and, therefore, the analysis with the more standard 8 mm kernel is reported in the article.
