# Supplementary material for: Amount, not strength of recollection, drives hippocampal activity: A problem for apparent word familiarity‐related hippocampal activation
Source: Hippocampus. 2018 Nov 8;29(1):46–59. doi: 10.1002/hipo.23031 (PMC6492455; doi:10.1002/hipo.23031)
Supplement: Supplementary file 1 — Supporting Information [file HIPO-29-46-s001.docx]

# Supplementary Material

**Article:** Amount, not strength of recollection, drives hippocampal activity: a problem for apparent word familiarity-related hippocampal activation

by Mayes, A. […] & Kafkas, A.

#
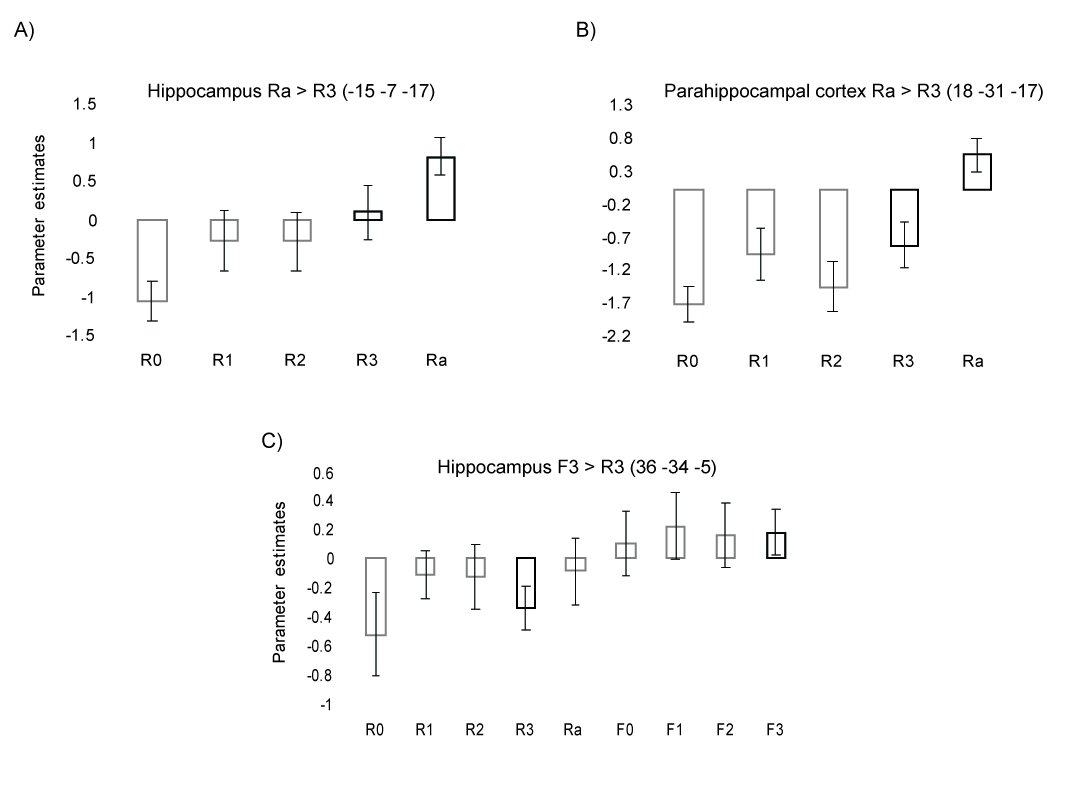
Supplementary Figure

Supplementary Figure 1. Additional parameter estimates plots linked to Figure 3 (A, B) and Figure 6 (C). Activation parameters are plotted for all the conditions within each task (A and B) or across both tasks (C) for the clusters of significant activations as reported in the manuscript. Conditions in black are the ones contrasted in the main analysis. Error bars show the standard error of the mean.

# Supplementary Tables

# Supplementary Table 1. Whole brain activations for additional (Ra) versus strong recollection (R3; Ra > R3)

| Side | Region | No. of Voxels | ~BA | MNI x y z | T-value |
| --- | --- | --- | --- | --- | --- |
| L | Angular Gyrus | 84 | BA 39 | -42 -58 31 | 7.15 |
| R | Parahippocampal Cortex | 16 | BA 35 | 18 -31 -17 | 10.86 |
| L | Hippocampus | 10 |  | -15 -7 -17 | 7.19 |
| L | Middle Temporal Gyrus | 20 | BA 21 | -45 -31 -2 | 6.44* |
| L | Middle Frontal Gyrus | 22 | BA 8 | -45 8 46 | 7.56* |
| L | Middle Occipital Gyrus | 29 | BA 19 | -36 -82 28 | 7.69* |
| R | Precuneus | 25 | BA 7 | 0 -67 49 | 6.04* |

*Note:* * *P* < 0.001, uncorrected. All the other effects are FWE-corrected at the cluster level.

Supplementary Table 2. Whole brain activations when comparing strong familiarity (F3), strong recollection (R3) and additional recollection (Ra)

| Side | Region | No. of Voxels | ~BA | MNI x y z | T-value |
| --- | --- | --- | --- | --- | --- |
| **R3 > F3** | | | | | |
| L | Middle Occipital Gyrus | 1172 | BA 19 | -30 -82 19 | 11.79 |
| L | Inferior Parietal Lobule |  | BA 40 | -27 -49 43 | 7.29 |
| R | Postcentral Gyrus | 91 | BA 2/3 | 39 -31 46 | 7.85 |
| L | Superior Frontal Gyrus | 451 | BA 6 | -21 -1 52 | 7.64 |
| R | Middle Occipital Gyrus | 825 | BA 19 | 33 -88 10 | 7.45 |
| R | Superior Parietal Lobule |  | BA 7 | 24 -55 58 | 6.7 |
| R | Middle Frontal Gyrus | 87 | BA 6 | 27 -4 49 | 6.72 |
| L | Inferior Frontal Gyrus | 43 | BA 45 | -33 32 25 | 6.62 |
| L | Middle Temporal Gyrus | 33 | BA 21 | -48 -52 4 | 6.33* |
| L | Ventral Lateral Thalamus | 96 |  | -12 -28 -2 | 5.93 |
| L | Putamen | 57 |  | -12 11 -8 | 5.92 |
| L | Rectal Gyrus | 57 | BA 47 | -12 23 -14 | 4.96* |
| R | Rectal Gyrus | 14 | BA 47 | 12 14 -14 | 4.96* |
| L | Lingual Gyrus | 23 | BA 18 | -12 -67 -2 | 4.61* |
| R | Middle Cingulate Gyrus | 10 | BA32 | 6 20 37 | 4.29* |
| **F3 > R3** | | | | | |
| L | Caudate Nucleus | 60 |  | -15 -4 22 | 7.34 |
| L | Angular Gyrus | 196 | BA 39/40 | -51 -67 34 | 7.42 |
| R | Angular Gyrus | 85 | BA 39/40 | 48 -64 37 | 6.91 |
| L | Postcentral Gyrus | 148 | BA 3/6/4 | -27 -37 64 | 6.85 |
| L | Superior Frontal Gyrus | 366 | BA8 /10 | -18 38 46 | 6.07 |
| L | Precuneus | 95 | BA 7 | 0 -64 28 | 5.16 |
| R | Postcentral Gyrus | 58 | BA3/6/4 | 21 -34 70 | 5.87* |
| R | Insula | 47 | BA 13 | 33 -22 4  45 -10 1 | 5.36* |
| L | Middle Frontal Gyrus | 25 | BA 8 | -33 32 43 | 4.5* |
| R | Hippocampus (posterior) | 10 |  | 36 -34 -5 | 3.68* |
| **Ra > F3** | | | | | |
| L | Fusiform gyrus | 184 | BA 37 | -48 -31 -20  -33 -67 -11 | 7.57 |
| L | Middle Occipital Gyrus | 45 | BA 19 | -30 -85 22 | 7.07 |
| R | Middle Temporal Gyrus | 12 | BA 37/19 | 51 -70 -2 | 6.82 |
| L | Inferior Parietal Lobule | 30 | BA 40 | -39 -40 49 | 5.95* |
| R | Fusiform Gyrus | 13 | BA 19/37 | 39 -70 -17 | 5.9* |
| R | Inferior Occipital Gyrus | 26 | BA 18 | 36 -91 -2 | 5.77* |
| L | Middle Frontal Gyrus | 22 | BA 6/9 | -24 2 49 | 5.73* |

*Note:* * *P* < 0.001, uncorrected. All the other effects are FWE-corrected at the cluster level.

# Supplementary Table 3. Individual participants analysis of Ra effects within the hippocampus for each of the 9 participants included in the random effect (group) analysis

| **Participant** | **peak Hippocampus MNI** | **Voxels** | **peak Hippocampus MNI** | **Voxels** |
| --- | --- | --- | --- | --- |
|  | **Ra > R3** | | **Ra > (R1+R2+R3)** | |
| P01 | 27 -16 -23  18 -21 -5 | 15 | 18 -31 -5  27 -16 -23 | 12 |
| P02 | -15 -7 -23  30 -13 -26 | 12 | -12 -7 -23 | 6 |
| P03 | -30 -34 -11 | 5 | -30 -28 -14  -30 -34 -11  -27 -16 -20 | 45 |
| P04 | 39 -16 -17  27 -22 -11  30 -25 -8  18 -25 -11 | 74 | -30 -31 -8  39 -16 -17  36 -28 -11 | 71 |
| P05 | 30 -40 4  -24 -25 -8  39 -31 -11 | 23 | -24 -25 -8  30 -40 4  36 -31 -11 | 21 |
| P06 | 42 -19 -17 | 9 | 39 -19 -17 | 14 |
| P07 | 30 -4 -23 | 11 | -18 -37 -2  27 -4 -23 | 10 |
| P08 | 36 -25 -17  36 -10 -14  24 -34 7 | 46 | 36 -10 -14 | 6 |
| P09 | 39 -13 -14  -30 -4 -29  -24 -25 -8 | 246 | 21 -37 1  -12 -40 7  24 -4 -23  -24 -4 -26 | 227 |

*Note:* All effects thresholded at *P* < 0.001, uncorrected

Familiarity and Recollection Instructions

Before we start the experiment, it is crucial that we carefully go through some instructions that will train you how to accurately identify the different types of memory you might experience. The two types of memory we are focussing on are recollection memory and familiarity memory, both of which may contribute to your ability to recognise a stimulus (e.g. a word) as something you have encountered previously.

**Recollection memory**

Sometimes when you are shown previously studied stimuli you will be able to recollect something specific about encountering them in the study session. In order to be recollecting you must be retrieving something that isn’t the stimulus that is in front of you or even part of it. Rather, you must be recalling something additional to the stimulus itself and this involves bringing to mind information that is not currently in front of you. The type of information you might recall includes what you were thinking when you encountered the stimulus during the study session. For example, you might have thought that a word was particularly funny, and this comes back to you when you see the word again during the memory test. You might also recall information that you might not have explicitly thought about during study, but also isn’t part of the stimulus. For example, you might recall that a stimulus came very early in the list or that you sneezed when the stimulus was originally presented.

However, if, when your memory is tested, you just remember seeing a stimulus or even part of a stimulus in the study session, but there is no other more specific extra information that you recall about the study session, then you should say that the stimulus is familiar (because you are recalling nothing about it that is specific to the study episode).

**Familiarity memory**

Everyone has a pretty good idea of what it is like to find a stimulus familiar without being able to recall anything about it. For example, we have all been in the situation where we have met someone whom we recognize as familiar, although we can’t recall anything about them such as their name, where we last saw them or why we know them.

You can have the same feeling of being sure that you encountered a stimulus in a specific context (such as a study session in a testing room) although you are unable to recall anything extra about what you thought or what else happened in the room when you encountered the stimulus. Sometimes the stimulus can feel very familiar and sometimes only weakly familiar.

The distinction between familiarity and recollection is very important in this experiment and it should not be confused with your feelings of confidence. You can be very confident that you have encountered something before, independent of whether you find it familiar or you recollect something about it.

Is the distinction clear to you?

Can you give the experimenter an example of when you have experienced feelings of familiarity?

Can you give the experimenter an example of when you have remembered something using recollection?

**Outline of experimental sequence presented to participants prior to commencing the study**
